# Tetracycline Enables Visualization of Remineralization Induced by Agents Penetrating Dental Enamel

**DOI:** 10.1007/s00223-026-01503-z

**Published:** 2026-04-22

**Authors:** Alexis Murat, Katia Jedeon, Anne-Lyse Denizot, Charlotte Duval, Gilles Richard, Sylvie Babajko, Sophia Houari

**Affiliations:** 1 UMR 1333 Oral Health, Team “Oro-facial Pathophysiology and Regeneration”, Université Paris Cité, Sorbonne Paris-Nord, INSERM, 1 rue Maurice Arnoux, Montrouge, 92120 France; 2 Septodont, Saint-Maur-des-Fossés, France; 3 Department of Restorative Dentistry and Endodontics, Rothschild Hospital, Assistance Publique- Hôpitaux de Paris (AP-HP), Paris, France

**Keywords:** Enamel, Dental varnish, Mineralization, *In vitro* quantification, Tooth

## Abstract

The demineralization of tooth enamel caused by acid attack in dental biofilms is a significant challenge in dentistry. While fluoride varnishes are widely used to protect and restore the enamel surface, their actual ability to penetrate and treat the enamel in depth remains poorly understood. This study proposes an innovative in vitro approach to visualize the diffusion of remineralizing agents into enamel depth using by confocal laser scanning microscopy (CLSM). Four commercial fluoride varnishes (MI Varnish^®^, Duraphat^®^, FluoroCal^®^, Clinpro Clear Fluoride^®^) were applied to artificially demineralized bovine enamel samples in the presence of tetracycline. We used the tetracycline’s properties to bind to calcium and form a fluorescent complex to track the remineralization activity of varnishes. The results demonstrate the absence of autofluorescence in the controls and the presence of a specific fluorescent emission for the active varnishes. MI Varnish^®^ demonstrated the most significant penetration in enamel depth (42.0 ± 11.6 μm), followed by Duraphat^®^, FluoroCal^®^, and Clinpro Clear Fluoride^®^. This non-destructive method provides accurate three-dimensional visualization of remineralized areas and is a promising tool for evaluating fluoride varnishes effectiveness.

**Supplementary Information:**

The online version contains supplementary material available at 10.1007/s00223-026-01503-z.

## Introduction

Tooth enamel is a complex multidimensional hierarchical structure composed of hydroxyapatite prisms and interprismatic spaces [1, 2]. This prismatic arrangement not only enhances enamel’s mechanical strength but also significantly influences the movement of ions within the tissue. The orientation of enamel prisms and the presence of interprismatic spaces create a network of microchannels that regulate the diffusion of water, ions, and acids [3, 4]. Enamel demineralization is a physicochemical process that occurs when the balance between mineral loss and gain at the tooth surface shifts toward net mineral loss. This phenomenon is primarily driven by acidogenic bacteria within the dental biofilm [5]. As these acids accumulate, the pH in the microenvironment drops below the critical threshold of approximately 5.5 [6], creating hydroxyapatite dissolution and porosities on enamel structure [7]. Clinically, enamel demineralization is characterized by opacity (white spot lesion), reduced fluorescence brightness and loss of enamel translucency [8]. The degree of porosity, the presence of trace elements and the orientation of prisms determine how quickly these processes occur, making enamel’s microstructure a critical factor in both the progression of demineralization and the effectiveness of remineralization strategies [9, 10].

Dentists currently treat demineralized enamel by remineralizing the surface with dental varnishes that contain high concentrations of calcium and phosphate, with or without fluoride [11, 12]. These varnishes typically contain a polymer that enables the retention of these ions at the enamel surface [13]. Agents for remineralizing human dental enamel include a wide range of products that aim to restore enamel mineral content and prevent carious lesions [14]. Among them, four different products are mainly used (Table 1): 1/ MI Varnish^®^ (GC) which contains sodium fluoride (NaF) 5% wt/vol and Casein PhosphoPeptide-Amorphous Calcium Phosphate (CPP-ACP) 2% wt/vol, 2/ Duraphat^®^ Fluoride varnish (Colgate) which contains 5% NaF, 3/ Clinpro Clear Fluoride^®^ (3 M) which contains 2.1% NaF calcium, phosphate, and 4/ FluoroCal^®^ (Bisco) which contains 5% NaF and Tri-Calcium Phosphate (TCP). All tested products are resin-based except Clinpro Clear Fluoride^®^ which is water-based. Nevertheless, limited comparative data exist on their ability to penetrate the lesion. In the literature, only the penetration of Icon^®^ infiltration resin into enamel has been investigated using rhodamine B fluorescence labeling visualized by confocal laser scanning microscopy (CLSM) [3]. In light of these observations, we propose a novel in vitro method for visualizing the phenomenon of varnish penetration in an artificially demineralized enamel model.

**Table 1:** Commercial products used in this study

Product	Manufacturer	Reference	Batch	Remineralizing agent (%)
MI Varnish^®^	GC	900,747	2,302,131	NaF (5%, wt/vol) + CPP-ACP (2%)
Fluorocal^®^	Bisco	B-30,507 K	BS6KM	NaF (5%, wt/vol) + TCP*
Duraphat^®^	Colgate	09VA019	33240BW9ZG	NaF (5%, wt/vol)
Clinpro Clear Fluoride^®^	3 M™	7,100,313,709	12,028,462	NaF (2,1% wt/vol)

*proportion not disclosed by the manufacturer

Tetracycline labelling, commonly used to evaluate remineralization in bone and dentin [15], is a chelator of metallic ions with a particular affinity for certain metal cations such as calcium [16]. When tetracycline binds to calcium ions, its generates a fluorescent calcium-tetracycline stable complex [17]. This approach has been widely used in several studies to measure bone mineralization in vitro and in vivo. When associated with calcein green or other fluorochromes, the use of tetracycline allows for the evaluation of the dynamics of bone mineralization in various species in vivo [18]. To a lesser extent, tetracycline has also been used to measure the dentine remineralization [19]. Enax and co-workers observed a significant tetracycline fluorescence along the dentin tubules following the application of a bioactive restorative cement highlighting in-depth mineralization.

A new procedure is proposed in this study to label the remineralization of enamel tested for comparative activities of mineralizing agents such as dental fluoride varnishes.

## Materials and Methods

### Experimental Design

Thirthy standardized blocks of bovine enamel (8 mm × 8 mm x 2 mm) (Intertek^®^ Company) were distributed into 5 groups (see Table 2 for the study design and products). As a vehicle, an internal varnish formulation (trade secret) was used without any active remineralization agents (TCP; CPP-ACP, fluoride). Each group, including MI Varnish^®^ (GC – 900747), Duraphat^®^ (Colgate – 09VA019), Clinpro Clear Fluoride^®^ (3 M – 7100313709), FluoroCal^®^ (Bisco – B-30507 K), and the vehicle was separated into two subgroups, with (test groups, four samples by product) or without tetracycline (control groups, two samples by product). For test groups, 1 gram of each varnish was mixed with 1% (w/w) tetracycline powder (Sigma-Aldrich) by hand-stirring for 2 min until a homogeneous varnish color was achieved.

**Table 2:** Study design

Study group	Tetracycline use	Product	Incubation media
0	No	Vehicle	Distilled water
1	No		Artificial saliva
	Yes		
2	No	Duraphat^®^	
	Yes		
3	No	Fluorocal^®^	
	Yes		
4	No	MI Varnish^®^	
	Yes		
5	No	3 M™ Clinpro Clear Fluoride^®^	
	Yes		

### Artificial Demineralization

To mimic early caries lesions, enamel blocks were subjected to an acid challenge. All enamel surfaces were oriented upward and immersed in a crystallizer containing 300 mL of 0.1 M lactic acid solution pH 4.4 (Sigma-Aldrich). The crystallizer was sealed with parafilm to prevent evaporation and placed in a climatic chamber at 37 °C for 72 h without stirring. The samples were then thoroughly rinsed with distilled water prior to varnish application.

### Varnish Application

All varnishes, containing tetracycline or not, were applied with a fine brush to ensure a thin and homogeneous layer covering the entire enamel surface, and dried for 5 min. In order to mimic the clinical situation, the varnished specimens were immersed for 4 h (as recommended by the manufacturers) in artificial saliva (pH 7, 37 °C with stirring at 130 rpm). The artificial saliva contained 150 mM potassium chloride, 1.5 mM calcium nitrate, and 0.9 mM potassium phosphate for groups 1 to 5 or distilled water for the group 0. Then, varnish layer was gently removed with wet damp cloth and all specimens were replaced into their corresponding incubating media (artificial saliva or distilled water). At the end of the incubation period, samples were rinsed in water and stored at 4 °C.

### Confocal Laser Scanning Microscopy Characterization of Cross-Sectional Enamel

Prior to analysis, each sample was cut longitudinally with a circular diamond saw (Komet dental, Germany) to obtain a cross-sectional view of the enamel. Cross-sections were examined with a Zeiss LSM 710 confocal laser scanning microscope (CLSM) equipped with a 405 − 30 diode laser. The excitation wavelength was set at 405 nm and the fluorescence emission was recorded at 490 nm according to the tetracycline fluorescent dye parameters. A three-dimensional image with a resolution of 512 × 512 × 30 pixels (x, y and z, respectively) of a representative area of each sample cross section was acquired. Fluorescence depth was measured using ImageJ software (version 1.45) by detection of white pixels, from the enamel surface to the dentin, at three distinct locations per image. Twelve depth values per condition were used to compare the penetration depths between all tested varnishes. To further characterize the spatial distribution of tetracycline fluorescence within the enamel, intensity profiles of the most representative areas were generated along the depths for each condition tested (*n* = 3 per groups).

### Statistical Analysis

The data represent 12 measurements averaged per condition, with three areas per sample and four samples per product. Statistical analysis was performed using one-way Analysis of Variance (ANOVA) for all experimental groups. All statistical procedures were performed at the α = 0.05 level of significance with MiniTab v22 (Minitab, Inc, USA).

## Results

### Fluorescent Labeling with Tetracycline

**Fig. 1:**
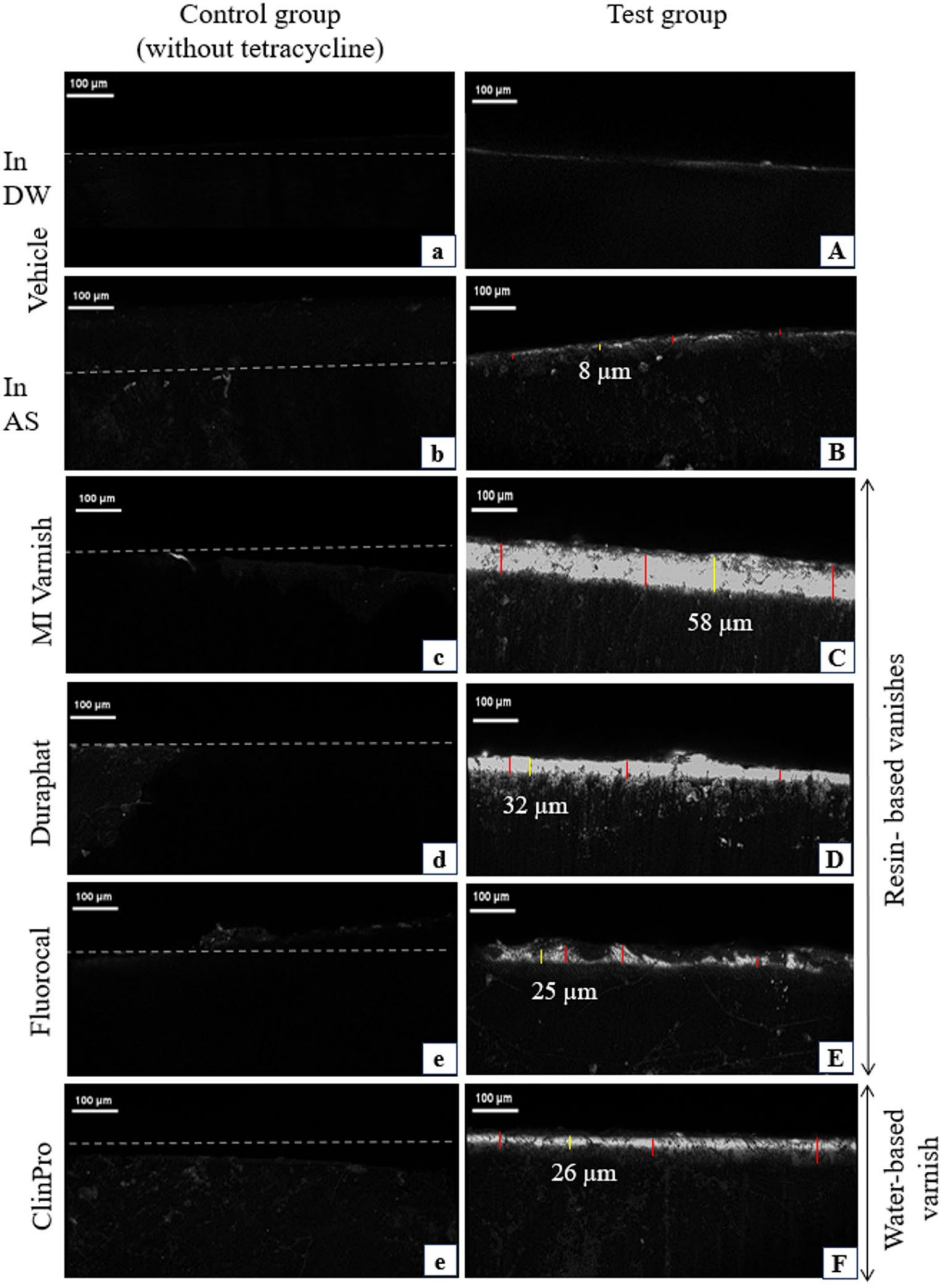
Confocal laser scanning microscopy (CLSM) visualization of tetracycline-induced fluorescence in demineralized bovine enamel after application of different fluoride varnishes. Cross-sectional CLSM images of bovine enamel treated with: (**a**, **A**) vehicle in distilled water; (**b**, **B**) vehicle in artificial saliva in distilled water; (**c**, **C**) MI Varnish^®^; (**d**, **D**) Duraphat^®^; (**e**, **E**) FluoroCal^®^; (**f**, **F**) Clinpro Clear Fluoride^®^. Images (**a**–**f**) correspond to control groups without tetracycline, showing no fluorescence. Images (**A**–**F**) represent test groups with tetracycline, revealing fluorescence corresponding to remineralized zones. Fluorescence intensity and penetration in depth depend on tested products. The red lines indicate where the depth measurements were taken for quantification while the yellow lines indicate the location for the fluorescence profiles. The dotted lines correspond to the surface of enamel. Scale bars, 100 μm

All test products applied to demineralized enamel without tetracycline (i.e. the negative control) showed no fluorescence on the enamel surface (Fig. 1a-f). These results indicate that there is no autofluorescence for any tested varnishes and of dental enamel. There was no fluorescence observed in the vehicle group (formula without active ingredients) immersed in distilled water (Fig. 1A). For the vehicle group immersed in artificial saliva with tetracycline, a slight fluorescence was observed at the enamel surface (Fig. 1B). The results of the control groups (dental varnishes without tetracycline) showed no fluorescence at the enamel surface after cross-section contrary to the test group with tetracycline incorporated into varnishes illustrating the specificity of fluorescence to labeling the enamel remineralization. Differences in fluorescence labeling were observed depending on the tested products (Fig. 1C–F). Representative fluorescence intensity of the enamel cross-section from each group are presented (Fig. 1). These results indicate that tetracycline specifically labels new remineralization area indicating mineralization agents’ ability to diffuse in enamel.

### Comparative Patterns of Fluorescence in Depth of Enamel

**Fig. 2:**
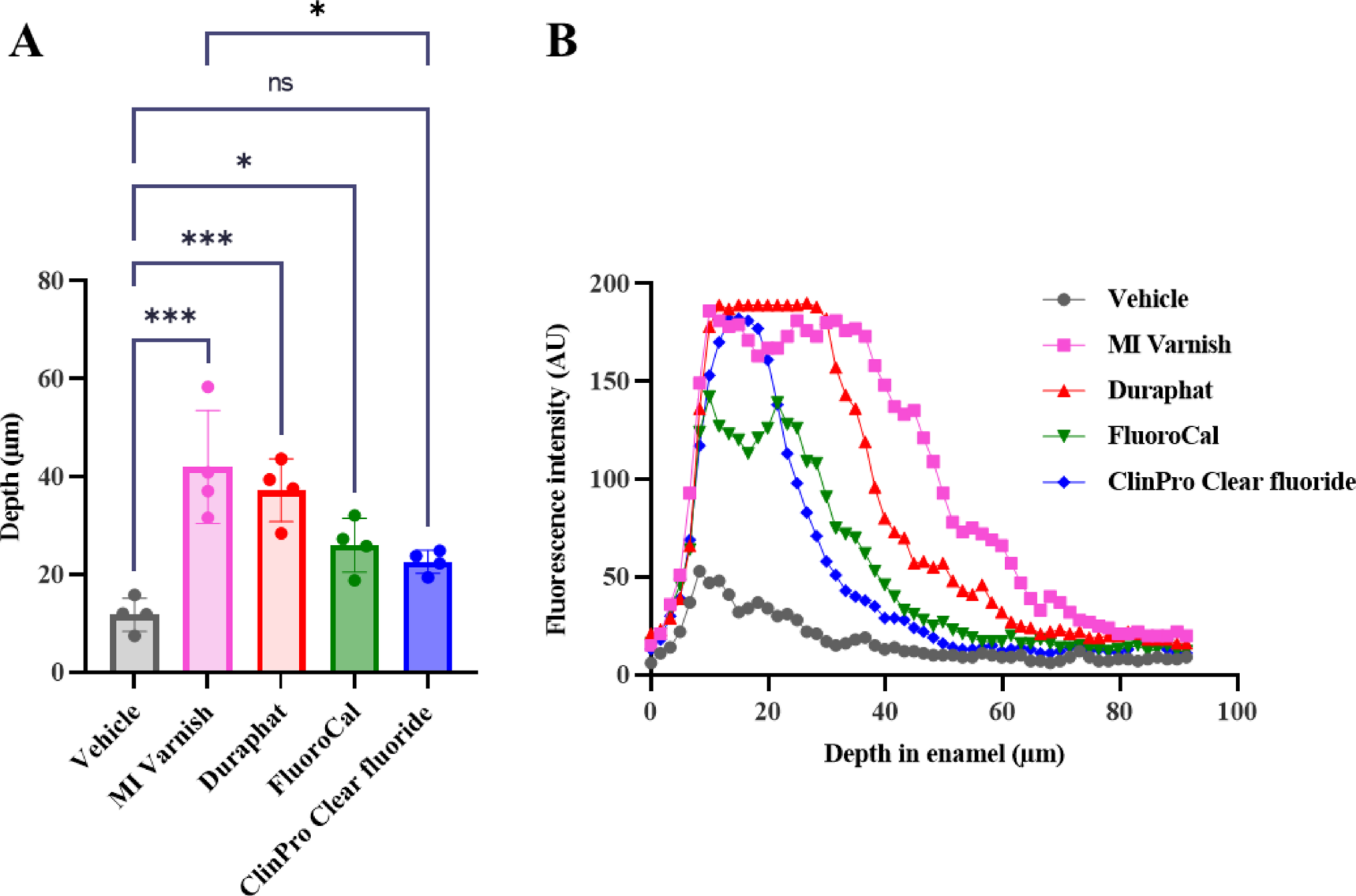
Comparative penetration depth and fluorescence intensity profiles of remineralizing agents among tested fluoride varnishes. **A** Mean fluorescence penetration depth (µm) measured by confocal laser scanning microscopy for each varnish: MI Varnish^®^ (42.0 ± 11.6 µm), Duraphat^®^ (37.2 ± 11.6 µm), FluoroCal^®^ (26.0 ± 5.5 µm), Clinpro Clear Fluoride^®^ (22.6 ± 2.4 µm), and vehicle in distilled water (11.8 ± 3.4 µm). Data represent the average of 12 measurements per condition (three measurements per sample, four samples per product). Inter-group comparison was carried out using one-way ANOVA test; significant differences were represented as * when *p* < 0.05, and *** when *p* < 0.001, **B** Graphs represent the fluorescence intensity with an arbitrary unit (AU) as a function of depth (µm) from the enamel surface for the most representative sample of each condition. These profiles illustrate the differences in remineralization patterns and penetration among the tested varnishes

Three representative areas for each sample (*n* = 4 samples by tested product) were measured based on CLSM images, and the mean fluorescence depth was calculated. Fluorescence images showed differences in homogeneity but also in depth between tested products (Supplementary Fig. 1). All varnishes, except ClinPro Clear Fluoride^®^, presented significantly deeper penetration compared to the vehicle (*P* < 0.05) (Fig. 2A). MI Varnish^®^ is the product which showed the deepest fluorescence (42.0 ± 11.6 μm), followed by Duraphat^®^ (37.2 ± 11.6 μm), FluoroCal^®^ (26.0 ± 5.5 μm) and ClinPro^®^ (22.6 ± 2.4 μm) (Fig. 2A). The comparison among groups showed a significant difference between MI Varnish and ClinPro Clear Fluoride (*p* < 0.05), no other significant differences were observed. Vehicle groups showed the shortest penetration depth (11.8 ± 3.4 μm).

The mean pattern of fluorescence in enamel depth was measured for all groups in similar conditions in three areas (Fig. 1, yellow lines) of the most representative sample (Fig. 2B). The pics of remineralization was observed at the surface of enamel, less than 40 μm in depth, for all tested products. Vehicle product demonstrated the lowest fluorescence penetration in term of both intensity (maximum 48.2 AU, the highest value) and depth. FluoroCal^®^ showed the lowest intensity compared to other tested products (139.2 AU). The three other products (MI Varnish^®^, Duraphat^®^, and Clinpro Clear Fluoride^®^) showed a maximum intensity of 190 AU, with Duraphat^®^ probably giving a more intense fluorescence than shown here because of the signal saturation. ClinPro promoted enamel mineralization on the surface of enamel whereas MI Varnish^®^ and FluoroCal^®^ penetrated into enamel more deeply.

## Discussion

### Tetracycline Fluorescence Follows Specifically Active Mineralizing Agents

Tetracycline’s strong affinity for calcium allows the fluorescent labeling of remineralization areas in enamel, which can be visualized in high resolution using CLSM. This approach allows the identification of the maximum intensity and the potential differences in remineralization patterns between varnishes. Compared to conventional methods such as surface microhardness testing or scanning electron microscopy (SEM), CLSM offers the advantage of non-destructive, three-dimensional imaging with high contrast and specificity when combined with fluorescent markers. While SEM provides superior morphological details and microhardness tests quantify mechanical changes, CLSM with tetracycline labeling enables dynamic visualization of mineralization processes in situ. Whereas tetracycline has been used to explore bone and dentin mineralization for many years, its application in enamel characterization hasn’t been reported to date. In this in vitro study, tetracycline was used to characterize remineralization provided by dental varnishes on artificial enamel lesions. The vehicle product (varnish without remineralizing agent) associated with tetracycline applied in distilled water (Fig. 1A) showed no fluorescence at any of the enamel cross-sections while a light fluorescence was observed on the surface sample immersed in artificial saliva (Fig. 1B). This observation highlights the role of saliva in enamel remineralization [20]. Indeed, saliva contains calcium and phosphorus, both forming hydroxyapatite crystals and contributing to enamel remineralization. However, this fluorescence labeling provided by artificial saliva stays much lower compared to dental varnishes (Fig. 1C–D).

### Varnish Properties Explaining Their Differential Remineralizing Abilities

For each tested product, a specific fluorescence signal directly related to its ability to remineralize and penetrate enamel depth was measured. A difference in penetration depths can be observed. MI Varnish^®^ and Duraphat^®^, both containing 5% NaF, showed the deepest penetration in enamel followed by Fluorocal^®^ and ClinPro Clear Fluoride^®^, the latter penetrating significantly less deeply into the enamel than MI Varnish^®^ and not significantly different than the vehicle (Fig. 2A). According to previous studies, MI Varnish^®^, containing CPP-ACP, is one of the most effective tested product [21]. Similarly, varnishes containing CPP-ACP (such as MI Varnish^®^) were shown to be more effective than the conventional varnishes with fluoride only such as with Duraphat^®^ used to prevent white spot lesions [22]. Indeed, our data suggest that Duraphat^®^ is probably more efficient than MI Varnish^®^ to induce remineralization at the surface of enamel, whereas the latter is able to diffuse deeper into enamel (Fig. 2B). MI Varnish^®^ contains CCP-ACP which binds calcium and thus favors remineralization and emission of fluorescence by tetracycline. These data need to be comforted and quantified by dose-response challenges. While the effectiveness of dental varnishes is well documented in the literature, the penetration of these products was investigated in few studies only. Moslehitabar and co-workers evaluated indirectly the enamel remineralizing effect of toothpastes containing CPP-ACP with and without fluoride or NaF alone, by Vickers microhardness analysis [23]. Authors report a similar remineralization efficiency for the 3 products on the depth window 0–50 μm and the highest effect beyond 50 μm maintained for CPP-ACP without fluoride, arguing that fluoride may interact with apatite at the surface of enamel and prevent the product from penetrating deeply into enamel. This aligns with the results obtained in the present study with varnishes containing low or no fluoride showing a lower mean depth fluorescence than others (MI Varnish^®^ *≥* Duraphat^®^ and Fluorocal^®^ *≥* ClinPro Clear Fluoride^®^). Thus, our data may be helpful for practitioners who may use the most appropriate varnish depending on the lesions to be treated. The remineralization effect beyond 50 μm may be too low to be identified by tetracycline fluorescence in CLSM, explaining why no fluorescence was observed beyond this distance. ClinPro Clear Fluoride^®^, the only water-based product tested in this study, exhibited the lower fluorescence depth, similar to Fluorocal^®^ varnish. The contact-time between the product and the enamel surface is not clearly reported in the literature. However, it is much lower than a resin-based varnish with resin as rosin which allows the varnish layer to last longer in situ [24]. Indeed, Constanza et al. reported that insoluble fluoride in varnish is leaching during intraoral exposure, increasing fluoride interaction with the enamel surface overtime [25]. Water-based varnish can be quickly solubilized in saliva, explaining why the fluorescence is lower than for other resin-based varnishes.

The ability of varnishes to penetrate defective enamel in depth is critical for their potency to treat dental caries and other acquired hypomineralized lesions that may be positioned in the depth of enamel as demonstrated by Houari et al. [26]. Fluoride, present in most formulas, has multiple extracellular beneficial effects for enamel remineralization and strengthening in topical application such as fluoridated apatite formation [27, 28]. That can explain why Duraphat^®^, one of the most described dental varnishes in the literature [29] with a high fluoride concentration (22,600 ppm), showed in this study a higher performance on intensity compared to Fluorocal^®^ and ClinPro Clear Fluoride^®^ (9,500 ppm). Other remineralizing agents, such as CPP-ACP in MI Varnish^®^ or TCP in Fluorocal^®^ act as a reservoir of calcium and phosphate ions, stabilizing them in amorphous form and releasing them at the tooth surface, facilitating the nucleation and reformation of hydroxyapatite [30].

### Limitations

This study has several limitations that should be considered when interpreting the findings. First, experiments were conducted on bovine enamel blocks, which differ structurally and chemically from human enamel [31], limiting direct clinical applicability. Moreover, the in vitro model does not replicate the complexity of the oral environment, including salivary flow, biofilm presence, temperature and pH fluctuations, and the patient’s tongue coming into contact with the treated area in an uncontrolled manner. In addition, the four-hour incubation time in artificial saliva may not reflect the real persistence of varnishes in the enamel surface in clinical conditions because of uncertain patient compliance and uncontrolled conditions of mouth’s patient (notably due to the contacts between treated teeth and the tongue). This study was focused on visualizing the penetration of remineralizing agents in enamel depth using fluorescence, without complementary analyses of mechanical, chemical and structural properties of enamel (microhardness, EDX, Raman). It could also be interesting to test enamel permeability-enhancing agents such as etching or hydrogen peroxide application in order to improve the dental varnish penetration and so the remineralization depth [32, 33]. Finally, despite standardized experimental conditions, total demineralization depths were estimated based on previous studies and not measured for each specimen.

### Conclusion

This study demonstrates that incorporating tetracycline into dental varnishes is an effective method for visualizing the penetration of remineralizing agents into demineralized enamel. CLSM imaging combined with tetracycline fluorescence provides a non-destructive and accurate tool for assessing the depth and distribution of remineralized areas. Despite the aim of this pilot study was not to compare the overall characteristics of commercialized varnishes, we observed that MI Varnish^®^ showed the greatest depth of penetration (that would be most suitable for deep lesions), followed by Duraphat^®^, FluoroCal^®^, and Clinpro Clear Fluoride^®^ (that would be sufficient for early surface lesions). These results pave the way for innovative approaches to compare the performance of dental varnishes and better understand their penetration performance. Although qualitative, the method reported here is a decisional tool that allows for choosing the best therapeutic option according to the lesion to be treated.

## Supplementary Information

Below is the link to the electronic supplementary material.

Supplementary Material 1
