# Supplementary material for: Tetracycline Enables Visualization of Remineralization Induced by Agents Penetrating Dental Enamel
Source: Calcif Tissue Int. 2026 Apr 22;117(1):58. doi: 10.1007/s00223-026-01503-z (PMC13102789; doi:10.1007/s00223-026-01503-z)
Supplement: Supplementary file 1 — Supplementary Material 1 [file 223_2026_1503_MOESM1_ESM.docx]

**Tetracycline enables visualization of remineralization induced by agents penetrating dental enamel**

**Supplementary data**

Alexis Murat (0009-0005-4645-407X)^1,2^, Katia Jedeon (0000-0002-6615-1464)^1,3^, Anne-Lyse Denizot^2^, Charlotte Duval^2^, Gilles Richard^2^, Sylvie Babajko (0000-0001-9047-3490)^1#^ and Sophia Houari (0000-0001-9450-0206)^1^

^1^Team « Oro-facial Pathophysiology and Regeneration », UMR 1333 Oral Health, Université Paris Cité, Sorbonne Paris-Nord, INSERM, Montrouge, France

^2^Septodont, Saint-Maur-des-Fossés, France

^3^Department of Restorative Dentistry and Endodontics, Rothschild Hospital, Assistance Publique- Hôpitaux de Paris (AP-HP), Paris, France

**#: corresponding author**

Sylvie Babajko, UMR 1333 Oral Health, 1 rue Maurice Arnoux, 92120 Montrouge, France

[Sylvie.babajko@inserm.fr](mailto:Sylvie.babajko@inserm.fr)

**Supplementary Fig.1**

**Further CLSM images of tetracycline-induced fluorescence in demineralized enamel after application of different fluoride varnishes**

Others samples used in this study for comparative depth analysis. The red lines indicate where the depth measurements were taken for quantification while the yellow lines indicate the location for the fluorescence profiles. Scale bars, 100 μm
